# The association between body mass index and severity of Coronavirus Disease 2019 (COVID-19): A cohort study

**DOI:** 10.1371/journal.pone.0247023

**Published:** 2021-02-16

**Authors:** Kulapong Jayanama, Sirawat Srichatrapimuk, Kanin Thammavaranucupt, Suppachok Kirdlarp, Supawadee Suppadungsuk, Thananya Wongsinin, Nithita Nanthatanti, Sithakom Phusanti, Dhanesh Pitidhammabhorn, Somnuek Sungkanuparph

**Affiliations:** Chakri Naruebodindra Medical Institute, Faculty of Medicine Ramathibodi Hospital, Mahidol University, Samut Prakan, Thailand; Azienda Ospedaliero Universitaria Careggi, ITALY

## Abstract

**Objectives:**

The coronavirus disease 2019 (COVID-19) has become a worst pandemic. The clinical characteristics vary from asymptomatic to fatal. This study aims to examine the association between body mass index (BMI) levels and the severity of COVID-19.

**Methods and study design:**

A cohort study included 147 adult patients with confirmed COVID-19 were categorized into 4 groups by BMI levels on admission: <18.5 (underweight), 18.5–22.9 (normal weight), 23.0–24.9 (overweight), and ≥25.0 kg/m^2^ (obese). Rates of pneumonia, severe pneumonia, acute kidney injury (AKI), and ICU stay during hospitalization across BMI group was determined. Logistic regression analysis was used to determine the association between BMI and severe pneumonia.

**Results:**

Of the totals, patients having a BMI <18.5, 18.5–22.9, 23.0–24.9, and ≥25.0 kg/m^2^ were 12.9%, 38.1%, 17.7%, and 31.3%, respectively. The rates of pneumonia and severe pneumonia tended to be higher in patients with higher BMI, whereas the rates of AKI and ICU stay were higher in patients with BMI <18.5 kg/m^2^ and ≥ 25 kg/m^2^, when compared to patients with normal BMI. After controlling for age, sex, diabetes, hypertension and dyslipidemia in the logistic regression analysis, having a BMI ≥25.0 kg/m^2^ was associated with higher risk of severe pneumonia (OR 4.73; 95% CI, 1.50–14.94; *p* = 0.003) compared to having a BMI 18.5–22.9 kg/m^2^. During admission, elevated hemoglobin and alanine aminotransferase levels on day 7 and 14 of illness were associated with higher BMI levels. In contrast, rising of serum creatinine levels was observed in underweight patients on days 12 and 14 of illness.

**Conclusions:**

Obesity in patients with COVID-19 was associated with severe pneumonia and adverse outcomes such as AKI, transaminitis and ICU stay. Underweight patients should be closely monitored for AKI. Further studies in body composition are warranted to explore the links between adiposity and COVID-19 pathogenesis.

## Introduction

The Coronavirus Disease 2019 (COVID-19), an infectious disease caused by severe acute respiratory syndrome coronavirus 2 (SARS-CoV-2), has become one of the worst pandemics in this century. The World Health Organization (WHO) announced the confirmation of COVID-19 as a pandemic on March 11^th^, 2020 [1]. As of May 26^th^, 2020, COVID-19 has affected over 5.5 million people worldwide, causing more than 347,000 fatalities [2]. The clinical outcomes of COVID-19 vary in severity from asymptomatic to lethal [3]. In addition to several degrees of pneumonia, COVID-19 may cause injury of many organs including liver, kidneys and heart [4].

Obesity, defined as excessive accumulation of body fat, is generally determined by body mass index (BMI), calculated by body weight (kg) divided by height squared (m^2^) [5]. The number of obese people is globally increasing. Adiposity affects adverse health outcomes such as coronary artery disease, cerebrovascular disease, insulin resistance, hypertension and fatty liver disease [6]. Fat accumulation does not only affect mechanical-related health complications, but the abundant adipose tissue also releases many adipokines which play a role in the inflammatory process [7]. Nonetheless, the immune system is suppressed in obese people, especially in vulnerable people with multiple comorbidities [8]. Obese people may be more susceptible to SARS-CoV-2 infection [9]. A pathophysiology of COVID-19 is an immune response dysfunction resulting in damage to multiple organs, particularly the lower airways [10]. Owing to similar pathogenesis, obesity could be correlated to adverse outcomes and severity of COVID-19.

Up to now, the data of the association between obesity and severity of COVID-19 is limited. The aims of this study were 1) to examine the characteristics of patients with COVID-19 across body mass index levels, 2) to assess the association between body mass index (BMI) and the severity of COVID-19, and 3) to evaluate the change of obese-related parameters across BMI levels during admission in patients with COVID-19.

## Materials and methods

### Patients and design

Human Research Ethics Committee, Faculty of Medicine Ramathibodi Hospital, Mahidol University approved the study. The approval number was MURA2020/947. Consent was not obtained because the data were analyzed anonymously. A cohort study was conducted in patients with confirmed COVID-19, aged 15 years and older, and admitted to Chakri Naruebodindra Medical Institute, Faculty of Medicine Ramathibodi Hospital, Mahidol University, Samut Prakan, Thailand, between March 12^th^ and April 30^th^, 2020. Diagnosis of COVID-19 was established by detecting SARS-CoV-2 RNA in nasopharyngeal swab specimens by real-time RT-PCR amplification of SARS-CoV-2 ORF1AB and N Gene fragments using a SARS-CoV-2 nucleic acid diagnostic kit (Sansure Biotech), which was approved by the National Medical Products Administration (NMPA) and certified by the China Food and Drug Administration (CFDA). All patients diagnosed with confirmed COVID-19 were admitted to the institute and analyzed.

Baseline demographic data including underlying diseases, risk exposures, and personal history were collected. Physical examinations including body weight and height were performed by trained physicians on the admission date. Blood tests and chest radiograph were also performed on admission. BMI was categorized into 4 groups: <18.5 kg/m^2^ (underweight), 18.5–22.9 kg/m^2^ (normal weight), 23.0–24.9 kg/m^2^ (overweight), and ≥25.0 kg/m^2^ (obese), following Asia-Pacific cut-off for underweight, overweight and obesity [11]. During hospitalization, blood tests were also corrected on day 7, 10, 12, and 14 of illness.

### Outcomes

Pneumonia was defined as clinical symptoms of respiratory tract infection together with abnormal lung imaging compatible with pneumonia. The patients with pneumonia were classified as severe pneumonia patients when having one of the following criteria: respiratory rate >30 breaths/min, severe respiratory distress, or an oxygen saturation ≤93% on room air [12]. Patients who had no symptom, mild symptoms, or non-severe pneumonia were described as having mild to moderate disease. Acute kidney injury (AKI) was defined as any of the following: increase in serum creatinine ≥0.3 mg/dL within 48 hours, increase in serum creatinine to ≥1.5 times from baseline, or urine volume <0.5 mL/kg/h for 6 hours [13]. Patients having an oxygen saturation <92% despite the use of an oxygen cannula 5 L/minute or respiratory rate ≥25 breaths/minute were admitted or transferred to intensive care unit (ICU). The rate of pneumonia, severe pneumonia, AKI, and ICU stay during hospitalization were determined in all patients.

### Statistical analysis

To describe the characteristics of patients with COVID-19 across BMI levels, baseline characteristics of the patients including demographic data, underlying diseases, and results of laboratory investigations are presented as mean ± standard deviation (SD) for continuous variables and as frequency (%) for categorical variables, stratified by BMI groups. The mean difference of continuous variables among BMI groups was tested using ANOVA. Categorical variables were analyzed using Chi-square test to determine the differences between groups. The rates of study outcomes (pneumonia, severe pneumonia, AKI and ICU stay) are illustrated in bar chart stratified by BMI levels.

To assess the association between BMI and the severity of COVID-19, predicting factors for severe pneumonia were analyzed using logistic regression models and presented using the odds ratios (OR) and the associated 95% confidence interval (CI). The potential predicting factors including age (years), sex, diabetes, hypertension, and dyslipidemia were used in the regression models as potential covariates. The two-way and three-way interactions of age and sex with BMI levels in relation to study outcomes were also tested in the regression models.

To evaluate the change of obese-related parameters across BMI levels in patients with COVID-19 during admission, the time-related changes of hemoglobin, creatinine, albumin, and alanine aminotransferase on day 7, day 10, day 12, and day 14 across the BMI groups are illustrated in line charts.

Statistical significance was considered as *p*< 0.05, and all reported probability tests were two-sided. The statistical analysis was conducted using IBM SPSS Statistics for Windows, Version 24.0 (Armonk, NY: IBM Corp).

## Results

This study included 147 patients diagnosed with confirmed COVID-19. The mean age of patients was 39.1±13.0 years and females were 59.5% of the patients. Percentage of patients having a BMI <18.5, 18.5–22.9, 23.0–24.9, and ≥25.0 kg/m^2^ were 12.9%, 38.1%, 17.7%, and 31.3%, respectively. More than half of the patients were active alcohol users. Hypertension and diabetes were common comorbidities. None of the patients with a BMI <18.5 kg/m^2^ had hypertension, whereas most patients with a BMI ≥23.0 kg/m^2^ had dyslipidemia. Patients in higher BMI levels tended to be older and had a higher proportion of males. Hemoglobin, creatinine and alanine aminotransferase levels significantly elevated in patients with higher BMI levels. Baseline characteristics on admission among patients with COVID-19 across BMI levels are presented in **Table 1**.

**Table 1:** Baseline characteristics of patients with COVID-19 on admission, categorized by body mass index levels.

Characteristics	Total *(N = 147)*	Body mass index (kg/m^2^)				*P* value
		< 18.5	18.5–22.9	23.0–24.9	≥25.0	
		*(N = 19)*	*(N = 56)*	*(N = 26)*	*(N = 46)*	
Age (years), mean ±SD	39.1±13.0	31.1±9.1	36.8±13.2	43.4±13.1	42.8±12.3	<0.001
Sex, male, number (%)	61 (41.5)	5 (26.3)	17 (30.4)	14 (53.8)	25 (54.3)	0.024
Underlying conditions, number (%)						
Diabetes	14 (9.5)	1 (5.3)	4 (7.1)	2 (7.7)	7 (15.2)	0.457
Hypertension	14 (9.5)	0 (0.0)	1 (1.8)	3 (11.5)	10 (21.7)	0.003
Dyslipidemia	8 (5.4)	0 (0.0)	0 (0.0)	1 (3.8)	7 (15.2)	0.005
Active smoking	30 (20.4)	4 (21.1)	13 (23.2)	5 (19.2)	8 (17.4)	0.335
Active alcohol drinking	81 (55.1)	10 (52.6)	33 (58.9)	14 (53.8)	24 (52.2)	0.957
Clusters of exposure, number (%)						0.067
Night club A	44 (29.9)	3 (15.8)	17 (30.4)	8 (30.8)	16 (34.8)	
Boxing stadium A	29 (19.7)	1 (5.3)	8 (14.3)	7 (26.9)	13 (28.3)	
Pub A	20 (13.6)	5 (26.3)	12 (21.4)	2 (7.7)	1 (2.2)	
Taxi driver A	7 (4.8)	1 (5.3)	4 (7.1)	1 (3.8)	1 (2.2)	
Others	47 (32.0)	9 (47.4)	15 (26.8)	8 (30.8)	15 (32.6)	
Education, number (%)						0.126
Less than secondary school	32 (22.4)	1 (5.3)	11 (20.4)	6 (23.1)	14 (31.8)	
Secondary school	65 (45.5)	10 (52.6)	27 (50.0)	8 (30.8)	20 (45.5)	
Bachelor degree	39 (27.3)	7 (36.8)	12 (22.2)	12 (46.2)	8 (18.2)	
More than bachelor degree	7 (4.9)	1 (5.3)	4 (7.4)	0 (0.0)	2 (4.5)	
Days of illness at admission (days), mean ±SD	6.9±4.1	8.7±5.6	7.0±4.0	6.2±4.1	6.4±3.3	0.161
Hemoglobin (g/dL), mean ±SD	13.5±1.7	12.8±1.2	13.0±1.5	13.8±1.5	14.1±1.8	0.002
Absolute lymphocyte count (/mm^3^), mean ±SD	1914.2±770.7	2044.1±832.2	1903.6±733.0	1880.50±567.3	1892.63±898.0	0.890
Creatinine (mg/dL), mean ±SD	0.81±0.23	0.71±0.14	0.76±0.22	0.83±0.14	0.90±0.28	0.003
Alkaline phosphatase (U/L), mean ±SD	67.3±40.8	73.4±86.8	57.4±14.6	72.7±39.9	73.7±31.7	0.158
Alanine aminotransferase (U/L), mean ±SD	31.0±24.1	22.4±14.4	24.45±13.7	26.9±21.2	45.0±32.2	<0.001
Total protein (g/dL), mean ±SD	78.2±5.8	75.1±6.8	78.3±6.1	78.7±4.8	79.1±5.1	0.076
Albumin (g/dL), mean ±SD	42.2±3.91	40.8±4.9	42.6±3.7	42.5±3.3	42.0±4.0	0.349

**Fig 1:**
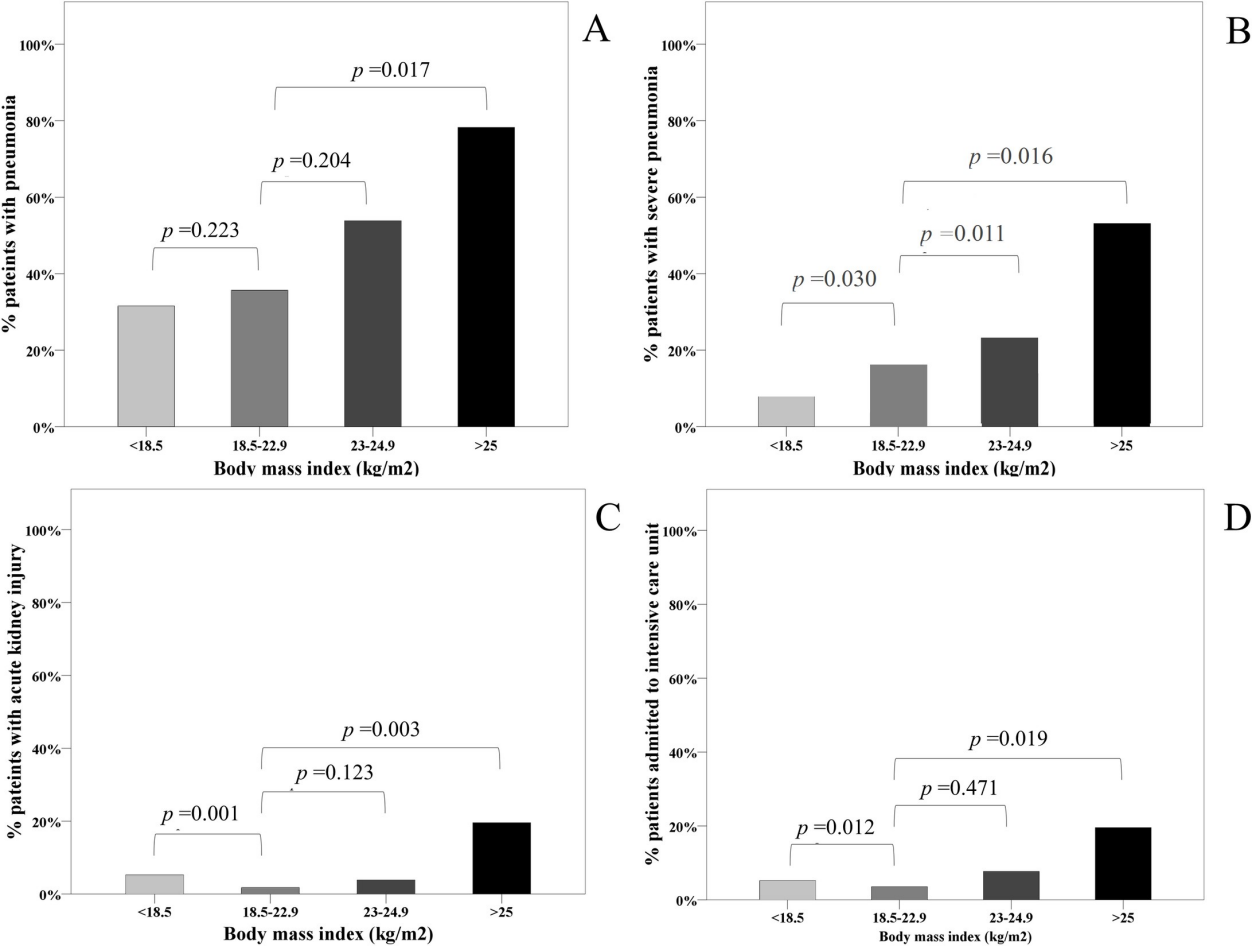
Rate of (A) pneumonia, (B) severe pneumonia, (C) acute kidney injury, and (D) ICU stay in each group of BMI levels.

Of the total patients, 76 patients (51.7%) had pneumonia and 20 patients (13.6%) had progressed to severe pneumonia. The rates of AKI and ICU stays were 8.2% and 9.5%, respectively. The rate of pneumonia tended to increase in patients with higher BMI levels; this rate in patients with BMI ≥25.0 kg/m^2^ was significantly higher (78.3%) when compared to patients with BMI 18.5–22.9 kg/m^2^ (35.7%) (**Fig 1**). The rate of severe pneumonia was significantly increased in each group of patients with higher BMI, i.e. 8.1%, 16.2%, 21.6% and 54.1% in patient with BMI < 18.5, 18.5–22.9, 23.0–24.9 and ≥25.0 kg/m^2^, respectively. The rates of AKI and ICU stay were higher in patients with BMI <18.5 kg/m^2^ and ≥ 25 kg/m^2^, when compared to patients with BMI 18.5–22.9 kg/m^2^ (**Fig 1**).

After controlling for age, sex, diabetes, hypertension and dyslipidemia in the logistic regression analysis, having a BMI ≥25.0 kg/m^2^ was associated with higher risk of severe pneumonia (OR 4.73; 95% CI, 1.50–14.94; *p* = 0.003) compared to having a BMI 18.5–22.9 kg/m^2^ (**Table 2**). Clinical characteristics of patients with severe pneumonia versus those with mild to moderate disease are summarized in **S1 Table**. The rates of pneumonia, severe pneumonia, AKI and ICU stay across the BMI groups are also shown in **S2 Table**.

**Table 2:** Association between body mass index and severe pneumonia, using multivariable-adjusted logistic regression analyses.

	Severe pneumonia			
	Model 1		Model 2	
	Beta-coefficient (95%CI)	*p*-value	Beta-coefficient (95%CI)	*p*-value
Body mass index (kg/m^2^)				
<18.5	2.73 (0.54–13.70)	0.224	2.56 (0.50–13.01)	0.257
18.5–22.9	reference		reference	
23.0–24.9	2.73 (0.76–9.83)	0.125	2.83 (0.77–10.36)	0.116
≥25.0	5.33 (1.76–16.12)	0.003	4.73 (1.50–14.94)	0.008

Model 1 was adjusted for age and sex.

Model 2 was adjusted for age, sex, diabetes, hypertension and dyslipidemia.

We did not find any two-way interactions of either age or sex with BMI levels in relation to pneumonia, severe pneumonia, AKI, and ICU stay, nor any three-way interactions of both age and sex with BMI levels in relation to pneumonia, severe pneumonia, AKI, and ICU stay.

**Fig 2:**
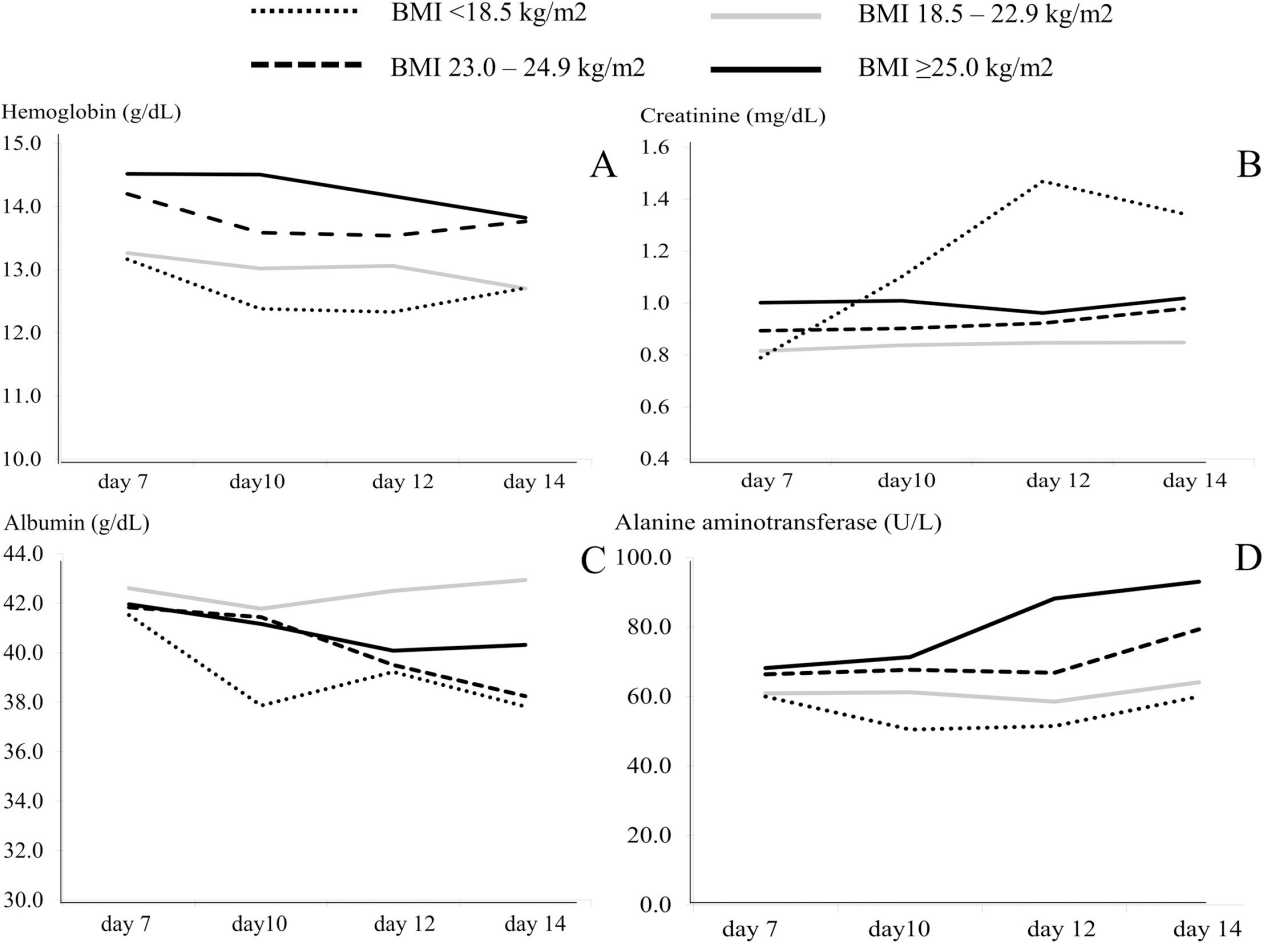
Time-related changes of (A) hemoglobin, (B) creatinine, (C) albumin, and (D) alanine aminotransferase, stratified by body mass index levels.

The mean of hemoglobin and albumin levels was significantly decreased from day 7 to day 14 of illness (*p*<0.001 and *p* = 0.04, respectively). Patients with higher BMI levels had higher hemoglobin and alanine aminotransferase levels during day 7 and day 14 of illness. Creatinine levels in patients having a BMI <18.5 kg/m^2^ tended to increase between day 7 and day 14 of illness and were significantly higher on day 12 and day 14 of illness, comparing with patients having a BMI ≥18.5 kg/m^2^ (*p*<0.05). Creatinine levels in patients having a BMI ≥25.0 kg/m^2^ tended to increase between day 14 and day 21 of illness and were significantly higher on day 21, comparing with patients having a BMI <25.0 kg/m^2^ (*p* = 0.019). **Fig 2** illustrates the time-related changes of hemoglobin, creatinine, albumin, and alanine aminotransferase on day 7, day 10, day 12, and day 14 in each group of BMI levels.

## Discussion

The present study included all patients with confirmed COVID-19 in Faculty of Medicine Ramathibodi Hospital, Mahidol University, during the study period. The majority of patients were middle aged. Approximately two thirds of the patients were either over or under normal weight. Nearly a half of the patients were diagnosed with pneumonia and a quarter of the patients with pneumonia had progressed to severe pneumonia. A higher BMI was significantly associated with severe pneumonia, after controlling for age, sex, diabetes, hypertension, and dyslipidemia.

The present study has demonstrated an increased rate of pneumonia and severe pneumonia in higher BMI levels. Moreover, obese patients (BMI ≥25 kg/m^2^) with COVID-19 were at higher risk of severe pneumonia, comparing to COVID-19 patients with normal weight. Similar to a previous study [14], our study demonstrated the dose response relationship between increase BMI and severity of COVID-19 focusing on severe pneumonia. The pathogeneses linked between obesity and severe COVID-19 could be immune dysregulation, comorbidities, and an impaired respiratory system [15]. Adiposity is related to immune dysregulation, including elevated inflammation and impaired host immune response. Fat cells, particularly visceral adipocytes, can induce macrophages to release interleukin (IL)-1, IL-6, IL-8, IL-10, tumor necrosis factor-α, c-reactive protein, and resistin. The overproduction of these proinflammatory cytokines, as a cytokine storm, is also a mechanism of lung injury as well as multiorgan failure in COVID-19 [16, 17]. Conceivably, chronic inflammatory state in obese people might be related to more severe inflammation and results in worsened outcomes in COVID-19. On the other hand, obese people are less adept at mounting immune response against microbes [8]. So SARS-CoV-2 and superimposed bacteria could infect and replicate more efficiently among this population. Additionally, patients having underlying medical conditions (e.g. serious heart conditions, diabetes, chronic kidney disease, etc.) might be at higher risk for severe COVID-19 [18, 19]. Obesity is associated with an increased risk of diabetes mellitus, hypertension, and cardiovascular disease [6]. The greater number of comorbidities in obese patients therefore increases the severity of COVID-19. The respiratory system is also changed in obese people. Altered respiratory mechanics, increased airway resistance, and decreased lung volume can impair gas exchange [20].

Our study revealed both underweight and obese patients with COVID-19 had a higher rate of AKI, after controlling for age, sex, diabetes, hypertension, and dyslipidemia. Both underweight and obese patients had increasing creatinine levels in the 2^nd^ or 3^rd^ week of illness. The causes of AKI in COVID-19 were proposed in various mechanisms including prerenal azotemia, acute tubular necrosis, direct viral injury, and thrombotic microangiopathy [21]. The increased severity of COVID-19 in obese patients related to higher rates of AKI. Severe pneumonia, acute respiratory distress syndrome (ARDS), and invasive mechanical ventilation use are predisposing factors of AKI [22]. As well, obesity-related inflammatory mediators could impair kidney function [15]. These pathogeneses might be an explanation to why the rate of AKI increased and the creatinine levels on the 3^rd^ week of illness were higher among obese patients with COVID-19, comparing with normal weight patients. Even so, the reason of creatinine level elevation among underweight patients with COVID-19 could differ. Patients with low BMI may be considered as malnutrition. The risk of AKI can be increased in malnourished patients when hospitalized [23]. Nausea, anosmia, and dysgeusia are common presenting symptoms of COVID-19 in the first period of illness, resulting in less appetite [24]. Reduced consumption, especially water, might result in dehydration and serum creatinine level increase on 2^nd^ week of illness in underweight patients with COVID-19.

This study presented patients with higher BMI levels had higher alanine aminotransferase levels both on admission and during hospitalization, comparing with patients with lower BMI level. The mechanisms of liver injury in patients with COVID-19 are inflammatory response, direct viral cytotoxicity, anoxia, and reactivation of pre-existing chronic liver disease [25]. Obesity is also related with chronic inflammation [26] and non-alcoholic fatty liver disease [27]. Viral hepatitis B and C profiles were tested in all patients with abnormal liver function test but none of the patients had HBV or HCV infection. The percentages of patients with active alcohol drinking were not different between different BMI levels. Abnormal alanine aminotransferase should be concerned in obese patients with COVID-19.

This is a well-designed prospective cohort. Since we included all patients with confirmed COVID-19 range from asymptomatic to fatal, the patients in this study are representative of all COVID-19 cases. This cohort followed until all patients clinically improved or died; therefore, the final outcomes were accurately determined and all data were completed when analyzed. Nevertheless, this study has some limitations that should be of concern. First, the sample size was modest; however, this study enrolled all adult patients from one of the largest cohorts in Thailand. Second, the treatment regimens were not evaluated in the analysis; nonetheless, all patients were treated as to the Thai National Guidelines recommended. Third, we did not assess the body composition of the patients due to the limited access to the patients during hospitalization.

### Conclusions

Obesity in patients with COVID-19 is associated with severe disease particularly severe pneumonia. The rates of adverse outcomes including AKI and ICU stay also increase in obese patients with COVID-19. The pathogenesis of AKI between underweight and obese patients may differ. Patients with a higher BMI are at higher risk for transaminitis. Further studies in body composition are warranted to explore the links between adiposity and severity of COVID-19.

## Supporting information

S1 Table Baseline characteristics on admission of patients with COVID-19, between mild to moderate disease and severe pneumonia. (DOCX)

S2 Table Rates of severe outcomes in patients with COVID-19, categorized by body mass index levels. (DOCX)

S3 Table Association of participants’ baseline characteristics with severe pneumonia, using univariate logistic regression analyses. (DOCX)

## Abbreviations

AKI: acute kidney injury
ARDS: acute respiratory distress syndrome
BMI: body mass index
COVID-19: Coronavirus Disease 2019
ICU: intensive care unit
IL: interleukin
SARS-CoV-2: severe acute respiratory syndrome coronavirus 2
SD: standard deviation
WHO: World Health Organization
