# Supplementary material for: The association between body mass index and severity of Coronavirus Disease 2019 (COVID-19): A cohort study
Source: PLoS One. 2021 Feb 16;16(2):e0247023. doi: 10.1371/journal.pone.0247023 (PMC7886119; doi:10.1371/journal.pone.0247023)
Supplement: S1 Table — (DOCX) [file pone.0247023.s001.docx]

**S1 Table. Baseline characteristics on admission of patients with COVID-19, between mild to moderate disease and severe pneumonia.**

|  | **Mild to moderate disease**  **(*N = 110*)** | **Severe**  **Pneumonia**  **(*N = 37*)** | ***p*-value** |
| --- | --- | --- | --- |
| Body mass index (kg/m^2^), number (%) |  |  | 0.001 |
| <18.5 | 16 (14.5) | 3 (8.1) |  |
| 18.5-22.9 | 50 (44.5) | 6 (16.2) |  |
| 23.0-24.9 | 18 (16.4) | 8 (21.6) |  |
| ≥25.0 | 26 (23.6) | 20 (54.1) |  |
| Age (years), mean ±SD | 36.3 ±11.7 | 47.5 ±13.2 | <0.001 |
| Sex, male, number (%) | 38 (34.5) | 23 (62.2) | 0.003 |
| Underlying conditions, number (%) |  |  |  |
| Diabetes | 7 (6.4) | 7 (18.9) | 0.024 |
| Hypertension | 5 (4.5) | 9 (24.3) | <0.001 |
| Dyslipidemia | 2 (1.8) | 6 (16.2) | 0.001 |
| Active smoking | 25 (22.7) | 10 (27.0) | 0.014 |
| Active alcohol drinking | 65 (59.1) | 16 (48.6) | 0.350 |
| Days of illness at admission (days), mean ±SD | 7.2 ±4.2 | 5.8 ±3.3 | 0.074 |
