# Supplementary material for: The association between body mass index and severity of Coronavirus Disease 2019 (COVID-19): A cohort study
Source: PLoS One. 2021 Feb 16;16(2):e0247023. doi: 10.1371/journal.pone.0247023 (PMC7886119; doi:10.1371/journal.pone.0247023)
Supplement: S2 Table — (DOCX) [file pone.0247023.s002.docx]

**S2 Table. Rates of severe outcomes in patients with COVID-19, categorized by body mass index levels**

| **Severe outcomes, number (%)** | **Total**  *(N =147)* | **Body mass index (kg/m^2^)** | | | | ***P* for trend** |
| --- | --- | --- | --- | --- | --- | --- |
|  |  | **< 18.5**  *(N =19)* | **18.5-22.9**  *(N =56)* | **23.0-24.9**  *(N =26)* | **≥25.0**  *(N =46)* |  |
| Pneumonia | 76 (51.7) | 6 (31.6) | 20 (35.7) | 14 (53.8) | 36 (78.3) | <0.001 |
| Severe pneumonia | 37 (25.2) | 3 (8.1) | 6 (16.2) | 8 (21.6) | 20 (54.1) | <0.001 |
| Acute kidney injury | 12 (8.2) | 1 (5.3) | 1 (1.8) | 1 (3.8) | 9 (19.6) | 0.008 |
| Intensive care unit stay | 14 (9.5) | 1 (5.3) | 2 (3.6) | 2 (7.7) | 9 (19.6) | 0.014 |
