# Supplementary material for: The association between body mass index and severity of Coronavirus Disease 2019 (COVID-19): A cohort study
Source: PLoS One. 2021 Feb 16;16(2):e0247023. doi: 10.1371/journal.pone.0247023 (PMC7886119; doi:10.1371/journal.pone.0247023)
Supplement: S3 Table — (DOCX) [file pone.0247023.s003.docx]

**S3 Table. Association of participants’ baseline characteristics with severe pneumonia, using univariate logistic regression analyses**

|  | **Severe pneumonia** | |
| --- | --- | --- |
|  | **Beta-coefficient (95%CI)** | ***p*-value** |
| Body mass index (kg/m^2^) |  |  |
| <18.5 | 1.56 (0.35-6.97) | 0.559 |
| 18.5-22.9 | reference |  |
| 23.0-24.9 | 3.70 (1.13-12.15) | 0.031 |
| ≥25.0 | 6.41 (2.29-17.92) | <0.001 |
| Age (per 1 year) | 1.07 (1.04-1.11) | <0.001 |
| Sex (male) | 3.11 (1.44-6.74) | 0.004 |
| Underlying conditions |  |  |
| Diabetes | 3.43 (1.12-10.56) | 0.031 |
| Hypertension | 6.75 (2.10-21.75) | 0.001 |
| Dyslipidemia | 10.45 (2.01-54.39) | 0.005 |
| Active smoking | 1.63 (0.35-1.49) | 0.153 |
| Active alcohol drinking | 0.73 (0.35-1.49) | 0.383 |
| Days of illness at admission (per 1 day) | 0.91 (0.82-1.01) | 0.076 |
